# Cisplatin-Resistant Gastric Cancer Cells Promote the Chemoresistance of Cisplatin-Sensitive Cells via the Exosomal RPS3-Mediated PI3K-Akt-Cofilin-1 Signaling Axis

**DOI:** 10.3389/fcell.2021.618899

**Published:** 2021-02-11

**Authors:** Meng-Yao Sun, Bo Xu, Qiu-Xue Wu, Wen-Lian Chen, Si Cai, Hui Zhang, Qing-Feng Tang

**Affiliations:** ^1^Department of Clinical Laboratory and Central Laboratory, Putuo Hospital, Shanghai University of Traditional Chinese Medicine, Shanghai, China; ^2^Cancer Institute, Longhua Hospital, Shanghai University of Traditional Chinese Medicine, Shanghai, China; ^3^Research Center for Traditional Chinese Medicine Complexity System, Institute of Interdisciplinary Integrative Medicine Research, Shanghai University of Traditional Chinese Medicine, Shanghai, China; ^4^Department of Clinical Laboratory, Jiading Branch of Shanghai General Hospital, Shanghai, China

**Keywords:** gastric cancer, cisplatin resistance, exosome, RPS3, PI3K-Akt-cofilin-1

## Abstract

Cisplatin is an important agent in first-line chemotherapy against gastric cancer (GC). However, consequential drug resistance limits its effectiveness for the treatment of GC. In this study, a cisplatin resistant gastric cancer cell line SGC7901R was determined by LC-MS/MS with increased exosomal levels of RPS3 protein. SGC7901R cell-derived exosomes were readily taken up by cisplatin-sensitive SGC7901S cells, thus triggering off a phenotype of chemoresistance in the receptor cells. Subsequently, it was demonstrated that exosomal RPS3 was essential for inducing chemoresistance of receptor cells as shown by the acquisition of this phenotype in SGC7901S cells with enforced expression of RPS3. Further mechanism study demonstrated that cisplatin-resistant gastric cancer cell-derived exosomal RPS3 enhanced the chemoresistance of cisplatin-sensitive gastric cancer cells through the PI3K-Akt-cofilin-1 signaling pathway. All these findings demonstrated that cisplatin-resistant gastric cancer cells communicate with sensitive cells through the intercellular delivery of exosomal RPS3 and activation of the PI3K-Akt-cofilin-1 signaling pathway. Targeting exosomal RPS3 protein in cisplatin-resistant gastric cancer cells may thus be a promising strategy to overcome cisplatin resistance in gastric cancer.

## Introduction

Gastric cancer ranks as the second most common malignant disease and the third leading cause of cancer-associated mortality in developing countries (Bray et al., 2018). Chemotherapy is one of the principal therapeutic approaches used for the treatment of gastric cancer. Chemoresistance has been identified as a major problem in the process of cancer treatment. Cisplatin (DDP) is widely used as a front-line chemotherapeutic agent for gastric cancer; however, chemoresistance limits the effectiveness of chemotherapy and results in treatment failure in the majority of cases (Guo et al., 2015; Zhang and Fan, 2017). In the past few years, some reports have partly shown the underlying mechanisms of cisplatin resistance in gastric cancer. However, the molecular mechanisms underlying cisplatin resistance remain to be further investigated.

Exosomes are a subset of extracellular microvesicles secreted from different types of cells (Zhang J. et al., 2015). More and more reports have indicated that exosomes play important roles in tumor growth, metastasis, angiogenesis, and immune regulation by acting as information communicators between different types of cells (Brinton et al., 2015; Meehan and Vella, 2016; Ruivo et al., 2017). Importantly, exosomes have recently been discovered anticipating in the regulation of chemoresistance in cancers (Sousa et al., 2015; Sharma, 2017; Butera et al., 2018). As is known to all, exosomes are nanosized vesicles (about 100 nm) loading a variety of kinds of cargo, including DNA, mRNA, miRNA, circRNA, and proteins (Colombo et al., 2014). Some reports have demonstrated that chemotherapy can change the exosomal composition secreted from tumor cells (Kreger et al., 2016; Ning et al., 2017; Bandari et al., 2018), however, which exosomal cargos play key roles in regulating chemoresistance is not well understood.

In this study, a cisplatin-resistant gastric cancer cell line SGC7901 (SGC7901R) was established, and the exosomal protein expression levels in SGC7901R cells and sensitive (SGC7901S) cells were investigated by LC-MS/MS and compared. RPS3 (ribosomal protein S3) was one of the highly expressed proteins in the exosome of SGC7901R cells in comparison with SGC7901S cells, which sparked our interest to explore the role and mechanism of exosomal RPS3 protein in transmitting a chemoresistance phenotype from cisplatin-resistant to cisplatin-sensitive gastric cancer cells.

It has been proposed that many ribosomal proteins (RPs) may act as cancer genes in human, such as high expression of RPL6 in human gastric cancer cell (Wu et al., 2011). RPL11 expression is a potential biomarker for predicting 5-FU sensitivity (Kawahata et al., 2020). Moreover, RPS15A could promote GC progression via activation of the Akt pathway (Liu et al., 2019). RPS3 has been demonstrated to be involved in DDP resistance of oral squamous cell carcinoma (Huang et al., 2019). However, the effects of RPS3 in DDP resistance of GC were unknown. Our previous study demonstrated that the PI3K/Akt pathway functions as a crucial pathway in the regulation of DDP resistance of GC by modulating PP1- and PP2A-mediated mitochondrial translocation of cofilin-1 (Tang et al., 2016). In addition, it was reported that RP mutations suppress the activity of the AKT pathway (Antunes et al., 2015). Therefore, we speculated that the mechanism of RPS3-related GC resistance to DDP may be associated with the PI3K/Akt signaling pathway, which finally induced PP1- and PP2A-mediated mitochondrial translocation of cofilin-1.

## Materials and Methods

### Establishment of Cisplatin-Resistant Cell Lines

The human gastric cancer cell lines SGC7901 and BGC-823 were obtained from the Cell Bank of Type Culture Collection of the Chinese Academy of Sciences (Shanghai, China). The cells were cultured in RPMI1640 medium (Gibco, Thermo Fisher Scientific, Inc.) supplemented with 10% fetal bovine serum (FBS, Gibco, Thermo Fisher Scientifics, Inc.) and incubated at 37°C in a humidified incubator with 5% CO_2_. Cisplatin-resistant SGC7901R and BGC823R cells were established by continuous exposure to stepwise-increasing concentrations of cisplatin (Sigma-Aldrich, Merck KGaA, Darmstadt, Germany). Cells that were viable in the cell culture medium with a high concentration of cisplatin (1,000 μg/L) were designated as cisplatin-resistant cells. Parental cells, denoted as SGC7901S and BGC-823S, were cultured under the same conditions without treatment.

### Exosome Isolation and Characterization

Gastric cancer cells were cultured in freshly prepared RPMI1640 medium containing exosome-free FBS for 48 h until cells had reached 90% confluence. Cell culture supernatants were collected and filtered using 0.22-μm pore filters (Merck KGaA), followed by differential centrifugation at 4°C: 300 × *g* for 10 min to remove cells, 2,000 × *g* for 15 min to remove cell debris, and 10,000 × *g* for 30 min to remove large particles. Then, the pellets containing exosomes were collected by spinning at 100,000 × *g* for 70 min. After washing with PBS, the pellets were dealt using ultracentrifugation (Beckman 70Ti rotor). The morphology of exosomes was examined via transmission electron microscopy. The number and size distribution of exosomes were detected by a LM10 nanoparticle characterization system (NanoSight, Malvern Instruments). The exosomal protein concentration was measured by the BCA method, and exosome-associated protein markers of HSP70, CD63, and CD9 expression were analyzed by Western blotting.

### Exosome Labeling and Treatment

8 × 10^4^ of SGC7901S cells were seeded in 12-well plates and incubated at 37°C with red fluorescent dye CM-Dil (Invitrogen; Thermo Fisher Scientific, Inc.) at 37°C for 1 h, washed with PBS, and centrifuged at 110,000 × *g* at 4°C for 70 min to remove excess dye. Exosomes from SGC7901R cells were pre-labeled with the green fluorescent dye PKH-67 (Invitrogen; Thermo Fisher Scientific, Inc.) at 37°C for 1 h, washed with PBS, and centrifuged at 110,000 × *g* at 4°C for 70 min to remove excess dye. Unlabeled exosomes were used as a negative control. The CM-Dil-labeled SGC7901S cells were incubated with PKH-67-labeled exosomes or unlabeled control exosomes for 4 h. Then, SGC7901S cells were fixed with 4% paraformaldehyde at room temperature for 1 h. Nuclear staining was performed with DAPI (40, 6-diamidino-2-phenylindole) at room temperature for 10 min. Incorporation of exosomes into targeted SGC7901S cells was visualized by fluorescence microscopy (Zeiss AG, Germany).

### Cell Proliferation Analysis

Cells were seeded in 96-well plates (5,000 cells/well) [MULTISCIENCES (LIANKE) BIOTECH, China] and exposed to increasing concentrations of cisplatin for 48 h at 37°C. The concentrations of cisplatin used for the drug dose-response curve analysis of cells were 0, 125, 250, 500, 1,000, 2,000, 4,000, 8,000, and 16,000 μg/L. The proliferative ability of cells was determined with a Cell Counting Kit-8 (CCK-8) (MedChemExpress, Monmouth Junction, United States) according to the manufacturer’s protocols. Exosomes were isolated from cells and cancer cells following transfection with oligonucleotides (described below). For exosome treatment analysis, each well in a 96-well plate was seeded with 1,000 cells and loaded with exosomes at 100 μg/mL for 48 h for functional analysis at 37°C, and the untreated cells served as the control. The cell culture medium was then removed, and a fresh medium containing the IC50 concentration of cisplatin was added to each well for 48 h. At the end of treatment, cell proliferation was measured.

### LC-MS/MS Analysis

20 μg proteins from each sample was denatured using 8 M urea, reduced with 10 mM dithiothreitol (DTT), and alkylated using 100 mM iodoacetamide. The samples were then proteolytically digested with endoproteinase LysC overnight at room temperature, followed by digestion with trypsin for 15 h at 37°C. The resulting peptide mixtures were extracted using a peptide extract solution (50% ACN, 0.1% TFA) for 30 min at 37°C. Then, the samples were dried and solubilized in the sample loading buffer containing 0.1% formic acid. Each sample of about 3–5 μg was analyzed by reversed-phase nano-liquid chromatography-tandem mass spectrometry (LC-MS/MS) (Thermo Scientific). The source data from three technical replicates of each sample were analyzed by searching the Uniprot human database with MaxQuant and Perseus software. Label-free quantitative (LFQ) values represent protein abundance. The false discovery rate (FDR) values at the protein and peptide levels were set to 1%. Only those proteins quantified in at least two out of three replicates in at least one group remain for further analysis. The multiple-sample ANOVA test was executed and corrected for multiple-hypothesis testing using a cutoff of FDR < 0.05.

### RT-qPCR

Total RNA from cells was extracted using TRIzol (Invitrogen; Thermo Fisher Scientific, Inc.). cDNA was produced by reverse transcription using the SuperScript^®^ III RT-PCR kit according to the manufacturer’s instructions (Thermo Fisher Scientific, Inc.). The amplification of fluorescence signals was detected by a fluorescence thermal cycler (Bio-Rad Laboratories, Inc., United States). RT-qPCR was performed (Novoprotein, Shanghai, China) using the following primers: RPS3 sense, 5′-GCGAGTTACACCAACCAGGA-3′ and antisense, 5′-ATGAACCGCAGCACACCATA-3′; β-actin sense, 5′-AGCAGCATCGCCCCAAAGTT-3′ and antisense, 5′-GGGCACGAAGGCTCATCATT-3′. B -Actin was set as an internal control for cellular mRNAs. The parameters for PCR quantification were as follows: 2 min at 95°C, followed by 40 cycles of 15 s at 95°C and 30 s at 60°C. The results of qPCR were defined using the quantification cycle (*C*q), and 2^–ΔΔ^*^*C*^*^*q*^ was used to calculate the relative expression levels (Livak and Schmittgen, 2001).

### Western Blotting

Cellular proteins were extracted with RIPA lysis buffer (Beyotime Institute of Biotechnology). Protein concentration was determined by the BCA method. A total of 40 μg of protein for each group was separated on 12% SDS-PAGE gels and transferred to 0.45-μm PVDF membranes (EMD Millipore). Membranes were then blocked with 5% BSA and incubated with primary antibodies at 4°C overnight against HPS70 (4876, CST), CD9 (ab92726, CST), CD63 (ab193349, Abcam), RBP4 (ab233138, Abcam), RPSA (ab133645, Abcam), RPS3(9538, CST), RPS20 (ab133776, Abcam), RPS14 (ab246916, Abcam), RPL4 (ab234829, Abcam), RPL13 (ab134961, Abcam), HSPD1 (ab46798, Abcam), HSPA8 (8444, CST), P-gp (13342, CST), p-cofilin-1 (3313, CST), cofilin-1 (5175, CST), PP1 (sc-7482, Santa Cruz), PP2A (9780, CST). Anti-β-actin (ab179467, Abcam) and anti-GAPDH (2118, CST) were used as the internal control. Anti-COX IV (11967, CST) was used as loading control for mitochondrial proteins. The membranes were then incubated at 37°C for 1 h with an HRP-conjugated secondary antibody (ab97051, Abcam). Bands were visualized by chemiluminescence according to the manufacturer’s protocols.

### Plasmid Construction and Cell Transfection

The recombinant plasmids containing the pcDNA3.1 empty vector, pcDNA3.1-RPS3, pLV4-shRNA/RPS3, and the non-targeting control pLV4-shRNA-negative control (NC) were produced by GeneChem, Inc. (Shanghai Genechem Company Ltd., China). All the transfections were performed using Lipofectamine 3000 reagent (Invitrogen; Thermo Fisher Scientific, Inc.) in accordance with the manufacturer’s instructions.

### ICP-MS Analysis

SGC-7901R or SGC-7901S cells were digested and collected. 500 μL ddH_2_O was added, and the cells were broken by freezing and thawing with liquid nitrogen repeatedly followed by sonication. The samples were centrifuged at 14,000 rpm/min for 10 min, and the supernatant was collected, dried under vacuum for 24 h, and dissolved in PBS. The dissolved samples were digested by 2 mL nitric acid followed by 1 mL perchloric acid, cooled down, set to a constant volume of 10 mL, and ultrafiltrated. The ultrafiltrated samples (10 mL) were diluted using a Gilson ASPEC XLi program to deliver 1.8 mL of iridium internal standard (0.005 mg/mL, in 1% nitric acid) and mixed thoroughly. Intracellular accumulation of DDP in each cell sample was determined by ultrasensitive multi-collector inductively coupled with mass spectrometry (ICP-MS) as previously described (Lavilla et al., 2009).

### Flow Cytometry Analysis

Cells were plated in 6-well plates at 2 × 10^5^ cells/well in RPMI 1,640 medium with 10% FBS. The cells were collected and re-suspended gently in 400 μL binding buffer. 5 μL Annexin V FITC was added to the above cell solution, gently vortexed, and incubated for 10 min at 4°C avoiding the light. 10 μL propidium iodide (PI) was added and cultured for another 5 min. Flow cytometry was then conducted using FACSCalibur Flow Cytometry (BD Biosciences, United States), and the results were analyzed with CellQuest software.

### Tumor Mouse Model

SGC-7901S cells were harvested in serum-free PBS, and 100 μL single-cell suspensions (2 × 10^7^ cells/mL) were injected into the subcutaneous area of male BALB/c nude mice (4–6 weeks old, SLAC Laboratory Lab, Shanghai, China). When the tumors reached an average size of 100 mm^3^, the mice were randomized into 5 groups (*n* = 5). Mice in group 1 were administered with normal saline daily, mice in group 2 were administered with 10 μg/mL DDP, and mice in groups 3, 4, and 5 proceeded administration of control exosomes, shRNA/RPS3 exosomes, and over/RPS3 exosomes, plus 10 μg/mL DDP, respectively. The length and width of tumors were recorded every 7 days. After 35 days, animals were sacrificed by cervical dislocation in deep anesthesia of CO_2_, and primary tumors were surgically removed and weighed. Tumor sizes were evaluated using the formula: length × width^2^ × 0.52. The primary tumors were analyzed by TUNEL assay, and p-PI3K, p-AKT, PP1, and PP2A proteins were detected by immunohistochemistry. All experimental protocols were reviewed and approved by the Animal Experimentation Ethics Committee of Shanghai University of Traditional Chinese Medicine.

### TUNEL Assay

TUNEL assay (a DeadEnd^TM^ Colorimetric TUNEL System kit, Promega Corp.) was performed to detect the apoptosis of the subcutaneous tumors in accordance with the manufacturer’s instructions.

### Immunohistochemistry (IHC)

Paraffin-embedded tumor tissue samples were selected for IHC. IHC for p-PI3K (ab182651, Abcam), p-AKT (4060, CST), PP1 (sc-7482, Santa Cruz), and PP2A (9780, CST) was carried out using a rabbit monoclonal or polyclonal primary antibody overnight at 4°C, an HRP-conjugated goat anti-rabbit IgG secondary antibody (1: 1,000, ab150077, Abcam) for 1 h at 37°C, and DAB staining solution (ab64238, Abcam) for 10 min at room temperature. The staining results were assessed with a Nikon E80i microscope (Nikon Corp.).

### Tissue Immunofluorescence

Dissected tissues were fixed in a mixture of 2% PFA and 20% sucrose solution for 24 h at room temperature and then embedded in Tissue-Tek O.C.T. (Electron Microscopy Sciences). Blocks were frozen in a dry ice and ethanol bath. For immunofluorescence, 6-μm O.C.T. tissue cryosections were stained with antibodies against P-cofilin-1 (3313, CST). Secondary antibodies conjugated to Alexa Fluor 594 (A-11032, 1: 1,000) were used (Life Technologies). Nuclear staining was done with DAPI (40, 6-diamidino-2-phenylindole). Immunofluorescence images were taken with fluorescence microscopy (Zeiss AG, Germany) (Qin et al., 2020).

### Statistical Analysis

All data are presented as the mean ± standard error of the mean from at least three independent experiments. A Student’s *t*-test, one-way analysis of variance, and a Tukey’s *post hoc* test were performed using GraphPad Prism 8.0 software. *P* < 0.05 was considered as statistically significant difference.

## Results

### SGC7901R Exosomes Induce a Cisplatin-Resistant Phenotype in SGC7901S Cells

**FIGURE 1:**
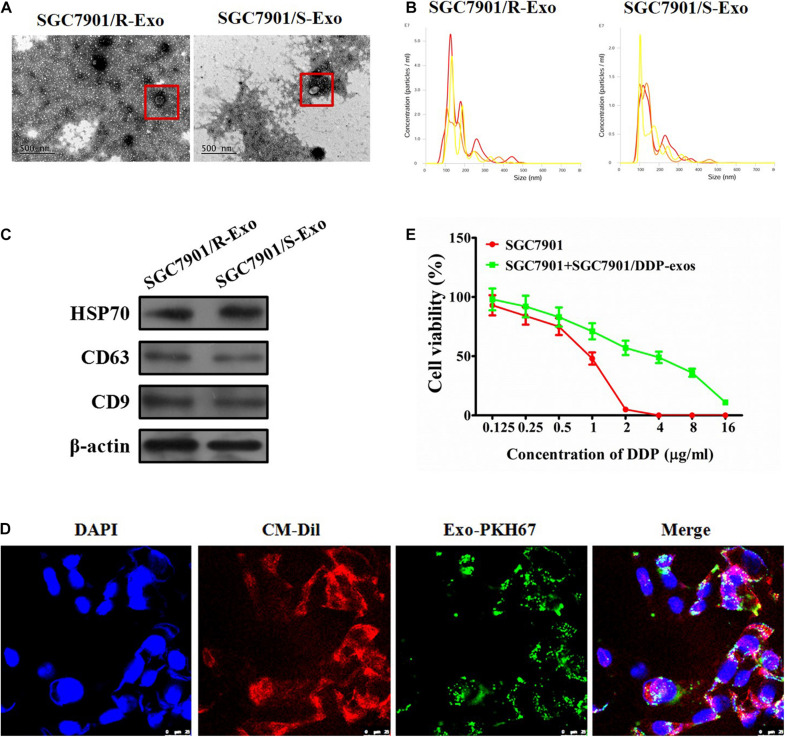
Effect of exosomes derived from SGC7901R cells on the proliferation of SGC7901S cells. **(A)** Exosome isolation from the cell culture medium of SGC7901R and SGC7901S cells was analyzed for the phenotype (purity and shape) by transmission electron microscopy. The red frames indicate the representative exosomes. Scale bar, 500 nm. **(B)** SGC7901R and SGC7901S cells were analyzed for size and particle number by the LM10 nanoparticle characterization system. Representative images were provided. **(C)** Western blotting was performed to detect the exosome markers, including HSP70, CD63, and CD9. β-Actin was used as an internal control. **(D)** Fluorescence imaging showed the delivery of PKH67-labeled exosomes (green) to CM-Dil-labeled SGC7901S cells (red). Cellular nuclei were stained with DAPI. Exosomes were derived from SGC7901R cells. Yellow arrows represented delivered exosomes in the representative images. Scale bar, 25 μm. **(E)** CCK-8 assay was performed to observe the effect of cisplatin (DDP) on the proliferation of indicated cells (SGC7901S cells or SGC7901S cells pretreated with exosomes derived from SGC7901R cells). The concentrations of cisplatin (DDP) used for the drug dose-response curve analysis of indicated cells was 0, 125, 250, 500, 1,000, 2,000, 4,000, 8,000, and 16,000 μg/L. Each experiment was performed in triplicate. All the data are shown as mean ± SD.

Several studies have demonstrated that chemoresistant tumor cells can release exosomes to chemosensitive tumor cells and transmit drug resistance during tumorigenesis (Kreger et al., 2016; Ning et al., 2017; Bandari et al., 2018). To analyze whether SGC7901R cell-derived exosomes may confer a malignant phenotype on cisplatin-sensitive tumor cells, exosomes from the culture medium of SGC7901R and SGC7901S cells were isolated. Purified exosomes from the cultures exhibited typical cup-shaped morphology by transmission electron microscopy analysis (Figure 1A). Using the LM10 nanoparticle characterization system, we detected the size and particle number of the purified exosomes. The mean size of SGC7901R exosomes and SGC7901S exosomes were both between 100 and 200 nm (Figure 1B), which corresponded with the reported size of exosomes (Kim et al., 2011). Moreover, the particle numbers for two tumor cell-derived exosomes were both more than 2.0 × 10^7^ particles/ml (Figure 1B). No significant difference in exosome quantities extracted from SGC7901R and SGC7901S cells was discovered. Next, the exosome markers were detected by Western blotting. Both of the two tumor cell-derived exosomes showed positive expression of exosome markers, including HSP70, CD63, and CD9 (Figure 1C).

To demonstrate that exosomes could be taken up by the recipient cells, CMDil-labeled SGC7901S cells (Red) were incubated with PKH67-labeled exosomes (green) derived from SGC7901R cells. In Figure 1D, we showed that PKH67 green fluorescence signals were visible around the nuclei and were also in the cytoplasm of SGC7901S cells following exposure to SGC7901R-derived exosomes. However, the negative control did not exhibit any green fluorescence (data not shown). This suggested the effective uptake of PKH67-labeled exosomes by SGC7901S cells. Subsequently, to investigate whether SGC7901R exosomes were responsible for the spread of chemoresistance, SGC7901S cells were incubated with SGC7901R exosomes for 48 h and then treated with DDP for chemosensitivity analysis. CCK-8 assays revealed that SGC7901R-exosome treatment significantly reduced the chemosensitivity of SGC7901S cells (Figure 1E). DDP had significantly lower inhibitory effects on SGC7901 + SGC7901/DDP-exos (IC50 = 3.145 μg/mL), relative to SGC7901 (IC50 = 0.8341 μg/mL) (*p* < 0.001). We used another gastric cancer cell line BGC823 to investigate whether BGC823R exosomes were responsible for the spread of chemoresistance. We detected BGC823R exosome treatment significantly reduced the chemosensitivity of BGC823S cells (Supplementary Figure 4).

### Cisplatin-Resistant Gastric Cancer Cell Line SGC7901R Exhibits Upregulated Levels of RPS3 Protein

**FIGURE 2:**
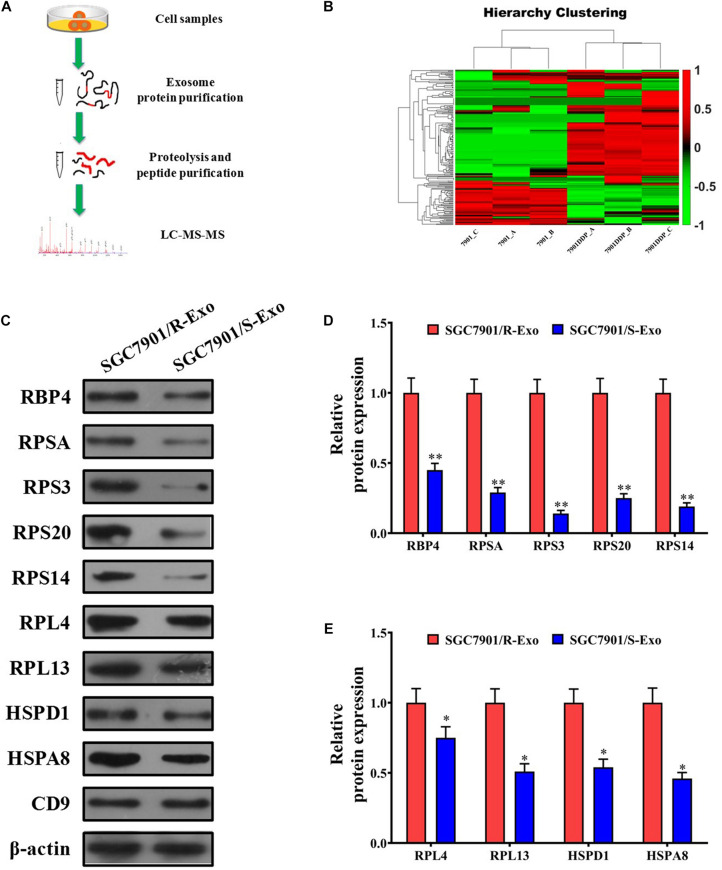
RPS3 was an important differentially expressed protein between SGC7901R cells exosomes and SGC7901S cells exosomes. **(A)** The schematic procedure of searching the differentially expressed proteins between SGC7901R cells exosomes and SGC7901S cell exosomes by LC-MS/MS analysis methods. **(B)** LC-MS/MS and cluster analysis of the differentially expressed proteins between SGC7901R cell exosomes and SGC7901S cell exosomes. Red represents high scores, and green represents low scores. The color brightness of each unit is associated with differences in multiples (log 2[AR/N]). **(C–E)** Western blotting was performed to validate the differentially expressed proteins in the exosomes of SGC7901R and SGC7901S cells. Each experiment was performed in triplicate. All the data are shown as mean ± SD. Student’s *t*-test was used to analyze the data. ^∗^*P* < 0.05, ^∗∗^*P* < 0.01 vs. SGC7901/R-Exo.

Since SGC7901R exosomes can induce the cisplatin-resistant phenotype of SGC7901S cells, it was very necessary to compare the contents in the exosomes of SGC7901R and SGC7901S cells. Although the exosomes contain a variety of kinds of cargo, including DNA, mRNA, miRNA, circRNA, and proteins, here in our study we just consider the protein contents that might play key roles in cisplatin-resistance delivery. By LC-MS/MS analysis methods (Figure 2A), we detected the differentially expressed proteins in SGC7901R cell exosomes and SGC7901S cells exosomes. The cluster analysis in Figure 2B demonstrated that many differentially expressed proteins were discovered between SGC7901R cell exosomes and SGC7901S cell exosomes. By GO function and KEGG pathway analysis, many important proteins were screened out, including RBP4, RPSA, RPS3, and RPS20 (Supplementary Figures 1–3). Next, Western blotting was performed to validate the significant differentially expressed proteins in the exosomes of SGC7901R and SGC7901S cells (Figures 2C–E). Considering that the difference was the most significant and acting as an important joint of the PPI network, as well as the preliminary experiments (data not shown), RPS3 was selected as the targets for further research. RPS3 protein level was also significantly upregulated in the exosomes of BGC823R cells when compared to BGC823S cells (Supplementary Figure 5).

### Exosomal Transfer of RPS3 Promotes the Cisplatin Chemoresistance in SGC7901S Cells

**FIGURE 3:**
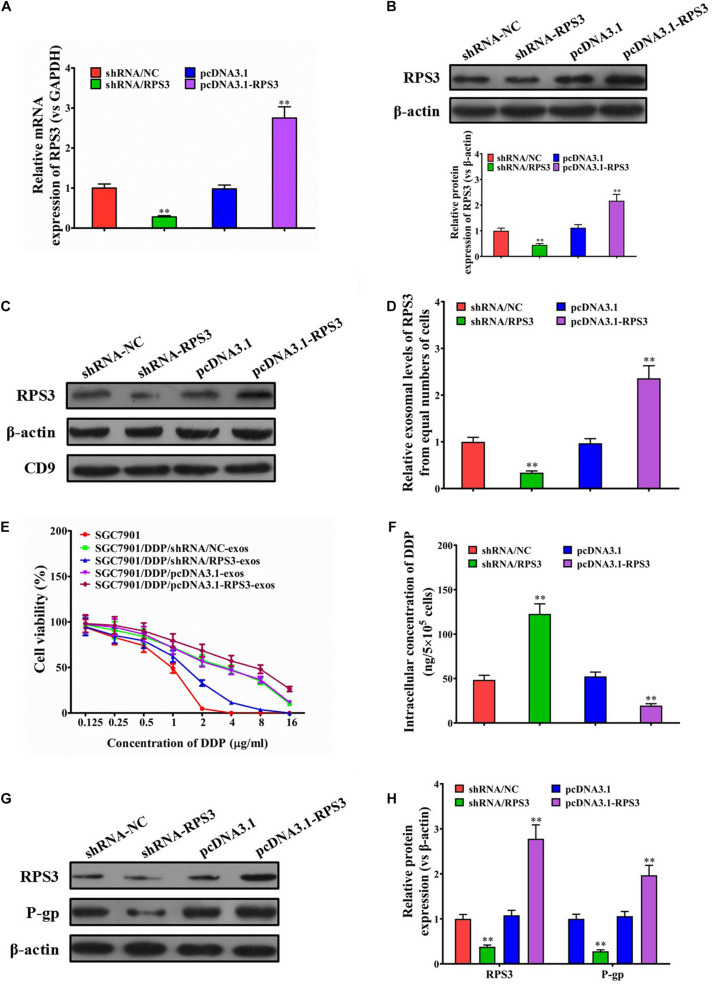
RPS3 overexpression confers DDP-resistant phenotypes from SGC7901R cells to SGC7901S cells. **(A)** RT-qPCR was performed to observe the effect of RPS3 knockdown and overexpression on the mRNA expression of RPS3 in SGC7901R cells. **(B)** Western blotting was performed to observe the effect of RPS3 knockdown and overexpression on the protein expression of RPS3 in SGC7901R cells. **(C,D)** Western blotting and quantitative analysis were performed to observe the effect of RPS3 knockdown and overexpression on the exosomal expression of RPS3 protein in SGC7901R cells. β-Actin was used as cellular control, and CD9 was set as the marker of exosomes. **(E)** CCK-8 assay was performed to observe the effect of cisplatin (DDP) on the proliferation of indicated cells: SGC7901S, SGC7901S + SGC7901R-shRNA/NC-exos, SGC7901S + SGC7901R-shRNA/RPS3-exos, SGC7901S + SGC7901R-pcDNA3.1-exos, SGC7901S + SGC7901R-pcDNA3.1-RPS3-exos. The concentrations of DDP used for the drug dose-response curve analysis of indicated cells were 0, 125, 250, 500, 1,000, 2,000, 4,000, 8,000, and 16,000 μg/L. Each experiment was performed in triplicate. **(F)** ICP-MS analysis was applied to see the effect of exosomes from indicated cells (SGC7901SR-shRNA/NC, SGC7901SR-shRNA/RPS3, SGC7901SR-pcDNA3.1, and SGC7901SR-pcDNA3.1-RPS3) on the DDP concentration in the cellular of SGC7901S cells. **(G,H)** Western blotting and quantitative analysis were performed to observe the effect of exosomes from indicated cells (SGC7901SR-shRNA/NC, SGC7901SR-shRNA/RPS3, SGC7901SR-pcDNA3.1, and SGC7901SR-pcDNA3.1-RPS3) on the P-gp protein expression in SGC7901S cells. β-Actin was used as cellular control. All the data are shown as mean ± SD. Student’s *t*-test was used to analyze the data. ^∗∗^*P* < 0.01 vs. SGC7901R-shRNA/NC.

To further validate that exosomal RPS3 is involved in the induction of a cisplatin-resistant phenotype, RPS3 was overexpressed or knockdown in SGC7901R cells (Figures 3A,B). Then, the exosomes derived from RPS3-overexpressing or -silencing SGC7901R cells were isolated. Western blotting results showed that RPS3 protein was enriched in exosomes derived from RPS3-overexpressing SGC7901R cells but downregulated in exosomes derived from RPS3-silencing SGC7901R cells (Figures 3C,D).

To evaluate whether the exosomal transfer of RPS3 promotes the DDP chemoresistance in SGC7901S cells, SGC7901S cells were pretreated with exosomes isolated from RPS3-overexpressing or -silencing SGC7901R cells. The results of CCK-8 assay revealed that the proliferation of SGC7901S cells was significantly reduced by RPS3-silencing SGC7901R cell exosome treatment but increased by RPS3-overexpressing SGC7901R cell exosome treatment following incubation with cisplatin (Figure 3E). Then, using ICP-MS for determination of the changed intracellular cisplatin concentration in SGC-7901S cells, we found that, when 6.73 μg/mL cisplatin exposed to above cells for 48 h, the intracellular accumulation of cisplatin increased in SGC7901S cells pretreated with RPS3-silencing SGC7901R cell exosomes but reduced in SGC7901S cells pretreated with RPS3-overexpressing SGC7901R cell exosomes compared with the control exosomes (Figure 3F). In addition, RPS3 and P-gp were both reduced in SGC7901S cells pretreated with RPS3-silencing SGC7901R cell exosomes but increased in SGC7901S cells pretreated with RPS3-overexpressing SGC7901R cell exosomes (Figures 3G,H). These results indicated that exosomal transfer of RPS3 protein may be involved in the induction of a cisplatin-resistant phenotype.

### RPS3-Rich Exosomes From SGC7901R Cells Decreased the Apoptosis of SGC7901S Cells Induced by Cisplatin via Affecting the Mitochondrial Translocation of Cofilin-1

**FIGURE 4:**
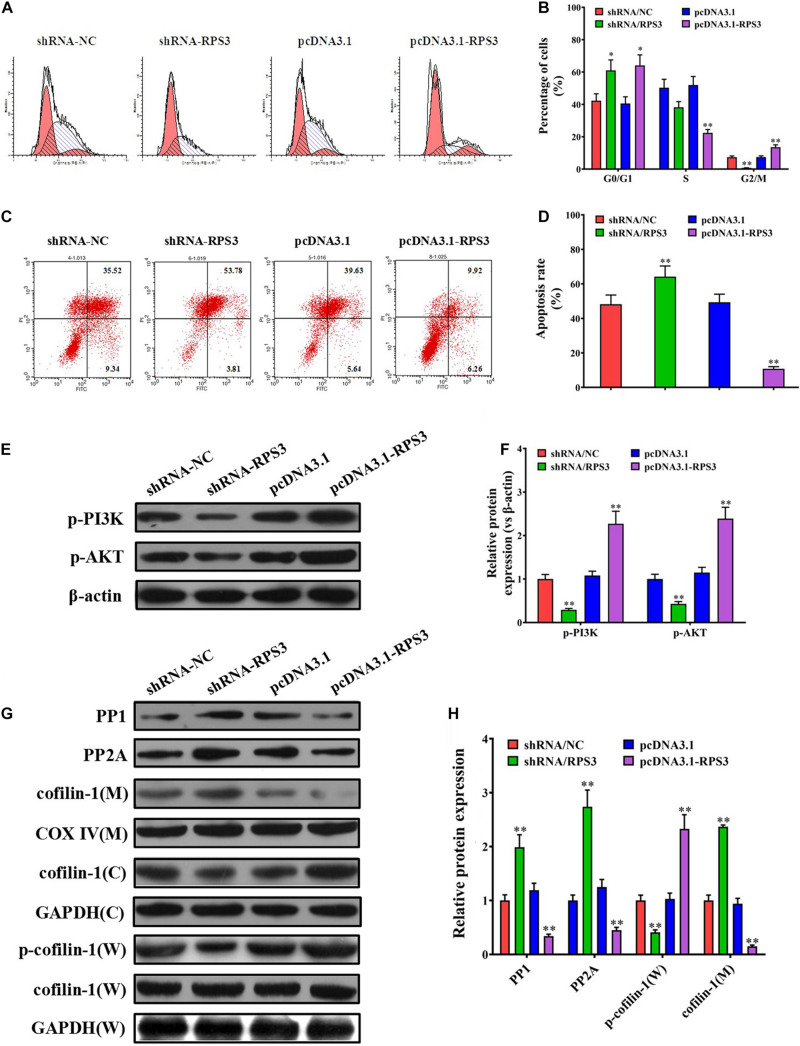
RPS3-rich exosomes from SGC7901R inhibited the apoptosis of SGC7901S via mitochondrial translocation of p-cofilin-1. **(A,B)** Flow cytometry method and quantitative assay were performed to observe the shift of cell cycles in SGC7901S cells treated with exosomes derived from RPS3-overexpressing or -silencing SGC7901R cells, or their respective controls. **(C,D)** Flow cytometry method and quantitative assay were performed to observe the change of cell apoptosis in SGC7901S cells treated with exosomes derived from RPS3-overexpressing or -silencing SGC7901R cells, or their respective controls. **(E–H)** Whole-cell lysates and mitochondrial and cytosolic proteins were prepared for the detection of p-PI3K, p-AKT, PP1, PP2A, p-cofilin-1, and cofilin-1 in the SGC7901S cells treated with exosomes derived from RPS3-overexpressing or -silencing SGC7901R cells, or their respective controls using western blotting. The levels of each protein were normalized against those of GAPDH (total and cytosolic proteins) or COXIV (mitochondrial proteins). Each experiment was performed at least in triplicate and all the data are shown as mean ± SD. Student’s *t*-test was used to analyze the data. ^∗^*P* < 0.05, ^∗∗^*P* < 0.01.

Flow cytometry was performed to see the effect of exosomes derived from RPS3-overexpressing or -silencing SGC7901R cells on the cell cycle and apoptosis of SGC-7901S cells following incubation with cisplatin. The results showed that pretreatment of exosomes derived from RPS3-silencing SGC7901R cells led to the reduction of SGC-7901S cells in G2/M phages compared with the control exosomes, while the exosomes derived from RPS3-overexpressing SGC7901R cells did the opposite effect (Figures 4A,B). In addition, pretreatment of exosomes derived from RPS3-silencing SGC7901R cells increased cisplatin-induced cell apoptosis of SGC-7901S cells compared with the control exosomes, while the exosomes derived from RPS3-overexpressing SGC7901R cells did the opposite effect (Figures 4C,D). Exosomes derived from RPS3-silencing BGC823R cells also induced cisplatin-related cell apoptosis in BGC823S cells compared with the control exosomes (Supplementary Figure 6). Furthermore, by Western blotting analysis, we showed that RPS3-rich exosomes from RPS3-overexpressing SGC7901R cells reduced the mitochondrial translocation of cofilin-1 in SGC-7901S cells compared with the control exosomes, while RPS3-reduced exosomes from RPS3-silencing SGC7901R cells did the opposite effect (Figures 4E–H). Immunofluorescence analysis also demonstrated that RPS3-rich exosomes from RPS3-overexpressing SGC7901R cells inhibited the translocation of cofilin-1 into the mitochondria (Supplementary Figure 8). These data implied that RPS3-rich exosomes from SGC7901R cells decreased the apoptosis of SGC7901S cells induced by cisplatin via affecting the mitochondrial translocation of cofilin-1.

### Overexpression of RPS3 Directly in SGC7901S Cells Exhibits Similar Effects to Treatment With SGC7901R Exosomes

**FIGURE 5:**
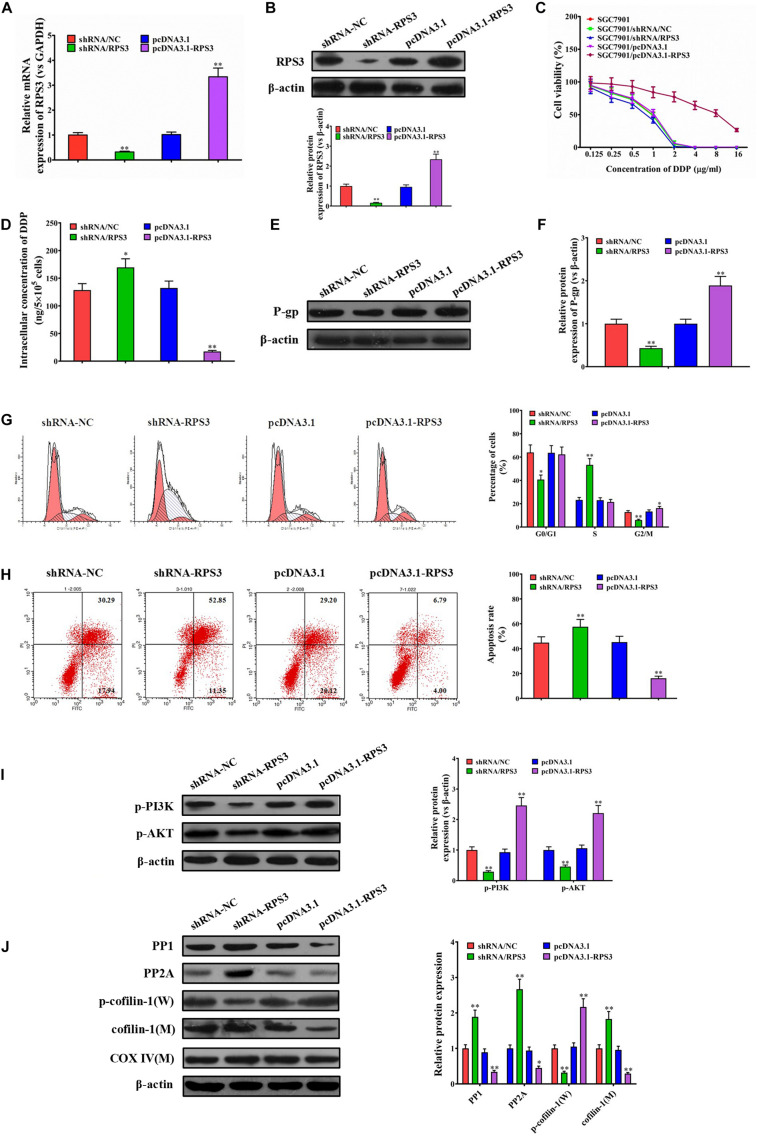
RPS3 overexpression directly in SGC7901S cells increases the DDP resistance of SGC7901S cells. **(A)** RT-qPCR was performed to observe the effect of RPS3 knockdown and overexpression on the mRNA expression of RPS3 in SGC7901S cells. **(B)** Western blotting was performed to observe the effect of RPS3 knockdown and overexpression on the protein expression of RPS3 in SGC7901S cells. **(C)** CCK-8 assay was performed to observe the effect of cisplatin (DDP) on the proliferation of indicated cells: SGC7901S, SGC7901S-shRNA/NC, SGC7901S + SGC7901S-shRNA/RPS3, SGC7901S-pcDNA3.1, and SGC7901S-pcDNA3. 1-RPS3. The concentrations of DDP used for the drug dose-response curve analysis of indicated cells were 0, 125, 250, 500, 1,000, 2,000, 4,000, 8,000, and 16,000 μg/L. Each experiment was performed in triplicate. **(D)** ICP-MS analysis was applied to investigate the effect of RPS3 knockdown and overexpression on the DDP concentration in the cellular of SGC7901S cells. **(E,F)** Western blotting and quantitative analysis were performed to observe the effect of RPS3 knockdown and overexpression on the P-gp protein expression in SGC7901S cells. β-Actin was used as cellular control. **(G)** Flow cytometry and quantitative assay were performed to observe the shift of cell cycles in SGC7901S cells treated with RPS3-overexpressing or -silencing, or their respective controls. **(H)** Flow cytometry and quantitative assay were performed to observe the change of cell apoptosis in SGC7901S cells treated with RPS3-overexpressing or -silencing, or their respective controls. **(I,J)** Western blotting and quantitative assays of p-PI3K, p-AKT, PP1, PP2A, p-cofilin-1, and cofilin-1 proteins in the SGC7901S cells treated with RPS3-overexpressing or -silencing, or their respective controls. The level of each protein was normalized against those of β-actin (total proteins) or COXIV (mitochondrial proteins). Each experiment was performed at least in triplicate, and all the data are shown as mean ± SD. Student’s *t*-test was used to analyze the data. ^∗^*P* < 0.05, ^∗∗^*P* < 0.01.

Our above data suggested that RPS3 expression levels were significantly elevated in SGC7901R cells, and SGC7901R-exosome treatment was proposed to confer cisplatin-resistant phenotypes to SGC7901S cells. To further investigate the role of RPS3 in the regulation of cisplatin chemoresistance, RPS3 was directly overexpressed or knockdown in SGC7901S cells. RT-qPCR and Western blotting analysis demonstrated that RPS3 was successfully overexpressed or knockdown in RPS3-overexpressing or -silencing SGC7901S cells (Figures 5A,B). Next, the CCK-8 assay revealed that the proliferation of SGC7901S cells was significantly reduced by RPS3 silencing but increased by RPS3 overexpressing following incubation with cisplatin compared with the controls (Figure 5C). Then, the ICP-MS analysis demonstrated that, when 6.73 μg/mL cisplatin was exposed to the above cells for 48 h, the intracellular accumulation of cisplatin increased in RPS3-silencing SGC7901S cells but reduced in RPS3-overexpressing SGC7901S cells compared with their controls (Figure 5D). Subsequently, we detected the protein expression of P-gp in RPS3-overexpressing or -silencing SGC7901R cells; the results showed that P-gp was reduced in RPS3-silencing SGC7901S cells but increased in RPS3-overexpressing SGC7901S cells compared with their controls (Figures 5E,F). These results indicated that overexpression of RPS3 directly in SGC7901S cells also exhibits similar effects to treatment with SGC7901R exosomes.

Next, flow cytometry results showed that knockdown of RPS3 directly in SGC7901S cells led to the reduction of SGC-7901S cells in G2/M phages, while the overexpression of RPS3 directly in SGC7901S cells did the opposite effect (Figure 5G). In addition, knockdown of RPS3 directly in SGC7901S cells increased cisplatin-induced cell apoptosis of SGC-7901S cells, while the overexpression of RPS3 in SGC7901S cells did the opposite effect (Figure 5H). Furthermore, RPS3-overexpressing SGC7901S cells reduced the mitochondrial translocation of cofilin-1, while RPS3-silencing SGC7901S cells did the opposite effect (Figure 5J). Additionally, we also found that RPS3-overexpressing SGC7901S cells reduced the expression of PP1 and PP2A and increased the expression of p-PI3K and p-Akt, while RPS3-silencing SGC7901S cells did the opposite effect (Figures 5I,J); these results were consistent with that in BGC823S cells (Supplementary Figure 7). These data suggested that RPS3 overexpression directly in SGC7901S cells decreased the apoptosis of SGC7901S cells induced by cisplatin via affecting the mitochondrial translocation of cofilin-1 and the expression of PP1 and PP2A, which were closely associated with PI3K-Akt signaling pathway. LY294002 was then used to inhibit the PI3K-Akt signaling pathway of SGC7901S cells. Western blotting analysis indicated that the RPS3 overexpression significantly inhibited cofilin-1(M) expression in SGC7901S. However, the levels of cofilin-1(M) did not noticeably change in LY294002-treated RPS3-overexpressed SGC7901S cells (Supplementary Figure 9), suggesting that RPS3 enhances the chemotherapy resistance of cells through the PI3K-AKt signaling pathway.

### Impact of RPS3-Rich Exosomes From SGC7901R Cells on the Growth of Subcutaneous Xenograft of SGC-7901S Cells in Nude Mice

**FIGURE 6:**
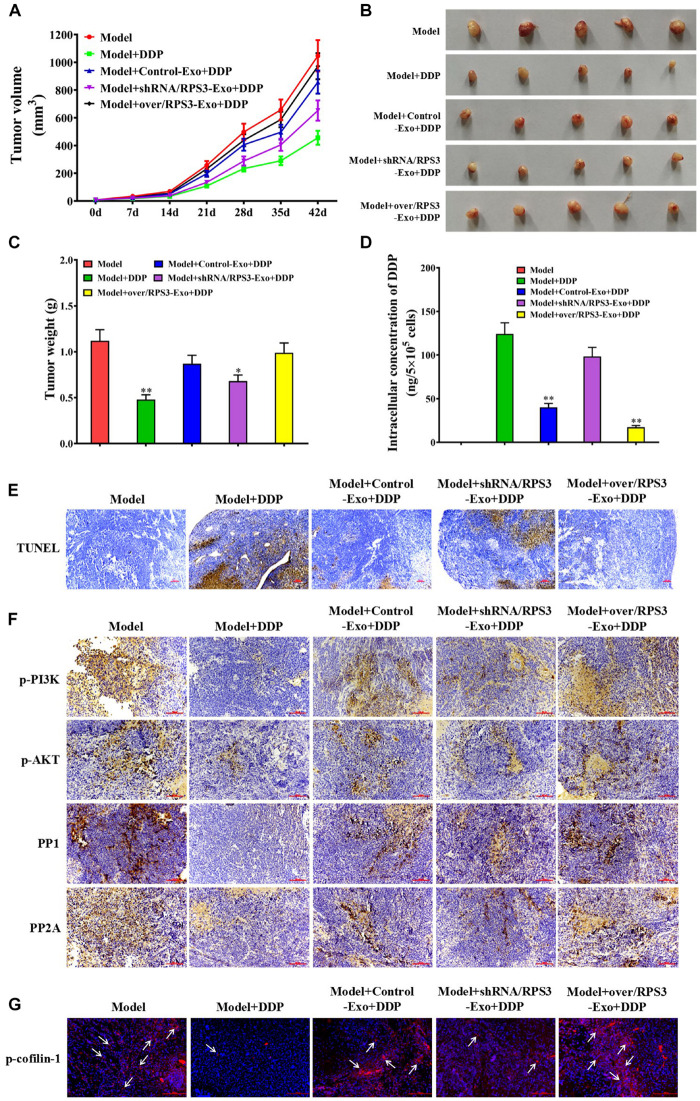
Impact of RPS3-rich exosomes from SGC7901R on the growth of subcutaneous xenograft of SGC-7901S. **(A)** Xenografts of SGC-7901S cells with indicated treatments performed on nude mice. SGC-7901S cells were pretreated with exosomes from RPS3-overexpressing or -silencing SGC-7901R cells, or control SGC-7901R cells. The length and width of the subcutaneous tumors were measured every 7 days. **(B,C)** Tumors were surgically removed from nude mice and weighed after administration for 35 days. ^∗^*P* < 0.05, ^∗∗^*P* < 0.01, in contrast to model groups treated with DDP alone. **(D)** ICP-MS analysis was applied to investigate the DDP concentration in the subcutaneous xenograft of SGC7901S cells with indicated treatments. **(E)** TUNEL assay was performed to observe the apoptosis of tumor cells in the subcutaneous xenograft of SGC7901S cells with indicated treatments. Scale bar, 100 μm. **(F)** The xenograft tumor tissues in mice of all groups were subjected to immunohistochemistry analysis using the antibody of p-PI3K, p-AKT, PP1, and PP2A. Scale bar, 100 μm. **(G)** Immunofluorescence detection of the expression of p-cofilin-1 in the subcutaneous xenograft of SGC7901S cells with indicated treatments. Scale bar, 100 μm. Each experiment was performed at least in triplicate and all the data are shown as mean ± SD. Student’s *t*-test was used to analyze the data. ^∗^*P* < 0.05, ^∗∗^*P* < 0.01.

To explore whether RPS3-rich exosomes from SGC7901R cells inhibited the pro-apoptotic effect of cisplatin *in vivo*, we employed a subcutaneous xenograft of SGC7901 cells in nude mouse models. As shown in Figure 6A, pretreatment of exosomes derived from RPS3-overexpressing SGC7901R cells reduced the inhibitory effect of cisplatin on the tumor growth in compared with cisplatin (0.6 mg/kg) alone, while the pretreatment of exosomes derived from RPS3-silencing SGC7901R cells did the opposite effect (Figure 6A). The final tumor resection and weight calculation also showed that pretreatment of exosomes derived from RPS3-overexpressing SGC7901R cells following incubation with cisplatin showed the highest tumor weight compared with cisplatin alone, while the pretreatment of exosomes derived from RPS3-silencing SGC7901R cells following incubation with cisplatin did the opposite effect (Figures 6B,C). Next, ICP-MS analysis was applied to investigate the cisplatin concentration in the subcutaneous xenograft of SGC7901S cells with indicated treatments. The results showed that pretreatment of exosomes derived from RPS3-overexpressing SGC7901R cells following incubation with cisplatin showed the highest levels of cisplatin concentration in the xenograft tumor compared with cisplatin alone, while the pretreatment of exosomes derived from RPS3-silencing SGC7901R cells following incubation with cisplatin did the opposite effect (Figure 6D).

In Figure 6E, pretreatment of exosomes derived from RPS3-overexpressing SGC7901R cells following incubation with DDP showed the least apoptotic tumor cells in the xenograft tumor compared with DDP alone, while the pretreatment of exosomes derived from RPS3-silencing SGC7901R cells following incubation with DDP did the opposite effect. Additionally, we detected the expression of p-PI3K, p-Akt, PP1, and PP2A in the xenograft tumor tissues by immunohistochemistry (IHC). The IHC images demonstrated that pretreatment of exosomes derived from RPS3-overexpressing SGC7901R cells following incubation with DDP showed the highest expression levels of p-PI3K and p-AKT and the lowest expression levels of PP1 and PP2A in the xenograft tumor compared with DDP alone, while the pretreatment of exosomes derived from RPS3-silencing SGC7901R cells following incubation with DDP did the opposite effect (Figure 6F). Finally, we detected the p-cofilin-1 expression by immunofluorescence in the subcutaneous xenograft of SGC7901S cells with indicated treatments. The results showed that pretreatment of exosomes derived from RPS3-overexpressing SGC7901R cells following incubation with DDP showed the highest expression levels of p-cofilin-1 in the xenograft tumor compared with DDP alone, while the pretreatment of exosomes derived from RPS3-silencing SGC7901R cells following incubation with DDP did the opposite effect (Figure 6G).

All the above data suggested that RPS3-rich exosomes from SGC7901R cells blocked the inhibitory effect of DDP on the growth of subcutaneous xenograft in nude mice by enhancing the cisplatin chemoresistance in SGC7901S cells.

## Discussion

Cisplatin has been widely used in tumor therapy, but the development of chemoresistance is often observed in patients with tumors (Guo et al., 2015; Zhang and Fan, 2017). At present, cisplatin resistance in tumors has been reported to be associated with underlying mechanisms, including increased drug efflux, alteration of intercellular signaling, tubulin mutation, and overexpression of β-tubulin isotype composition (Kavallaris, 2010; Vergara et al., 2012). Recently, it has been demonstrated that drug-resistant cancer cells could transmit chemoresistant phenotypes to chemosensitive cancer cells via exosomes (Sharma, 2017). Whether the acquisition of cisplatin resistance by gastric cancer cells occurs in this way remains unknown.

Among the main components in exosomal cargo, some molecules have been discovered acting as the vital regulating effect on chemoresistance (Cui et al., 2013; Soldevilla et al., 2014; Liu et al., 2016; Zeng et al., 2017). Exosomal miRNAs, lncRNAs, mRNA, and proteins could be shuttled from chemoresistant to chemosensitive cancer cells to transmit chemoresistance. For example, exosomal miR-222-3p from the gemcitabine-resistant lung cancer line A549 enhanced gemcitabine resistance in parental sensitive cells (Wei et al., 2017). Qu et al. (2016) identify lncARSR as a mediator of sunitinib resistance in renal cell carcinoma by acting as a competing endogenous RNA for miR-34 and miR-449, thereby increasing the expression of their targets AXL and c-MET. Exosomes from glioblastoma cancer cells harboring a protein tyrosine phosphatase receptor type Z1-MET fusion conferred temozolomide resistance on parental cells (Zeng et al., 2017). These findings demonstrated that exosomes could transfer intercellular drug resistance from drug-resistant to drug-sensitive cancer cells.

To investigate the mechanism of cisplatin resistance in gastric cancer cells, the cisplatin-resistant gastric cancer cell line SGC7901R was established in the present study. SGC7901R cell-derived exosomes could be effectively taken up by cisplatin-sensitive SGC7901S cells, and the receptor cells exhibited remarkably chemoresistance phenotypes. Consistently, it was demonstrated that SGC7901R-derived exosomes conferred a cisplatin-resistant phenotype in SGC7901S cells. Presently, a large number of differentially expressed molecules in the exosomes are discovered by RNA sequencing or proteomics, many of which are potentially associated with chemoresistance; however, quite a lot of them are little investigated.

In this study, cisplatin-resistant gastric cancer cell line SGC7901R was determined by LC-MS/MS with increased exosomal levels of RPS3 protein. RPS3, a component of the 40S ribosomal small subunit, is mainly involved in ribosomal maturation and initiation of translation (Schafer et al., 2006). RPS3 has various extra-ribosomal functions, including cell signaling (Kim et al., 2011), transcriptional regulation (Wan et al., 2007), and DNA repair (Kim et al., 2013). In addition, both upregulation and intrinsic dysfunctions in ribosomes result in an increased incidence of tumors, and RPS3 is involved in radioresistance or invasion of tumor cells (Kim et al., 2007; Kool et al., 2008). Huang et al. (2019) showed that Vitamin D promotes the cisplatin sensitivity of oral squamous cell carcinoma by inhibiting LCN2-modulated NF-κB pathway activation through RPS3. Zhao et al. (2019) unveiled a novel extra-ribosomal role of RPS3 in facilitating hepatocarcinogenesis via the posttranscriptional regulation of SIRT1 expression and proposes that the RPS3/SIRT1 pathway serves as a potential therapeutic target in HCC. A previous study showed that RPS3 was secreted into the extracellular environment in a dimeric form. All the reported data suggested that RPS3 may be a putative marker for malignant tumors.

Our data demonstrated that RPS3 protein was enriched in SGC7901R exosomes and could be delivered into SGC7901S cells. Overexpression of RPS3 directly in SGC7901S cells can also result in similar phenotypic effects as treatment with SGC7901R exosomes, which are then capable of inducing the malignant phenotype in the sensitive cells. A further mechanism study demonstrated that cisplatin-resistant gastric cancer cell-derived exosomal RPS3 could enhance the chemoresistance through the PI3K/Akt-mediated mitochondrial translocation of cofilin-1. The suppression of PI3K/Akt (LY294002) dramatically attenuated RPS3-induced DDP resistance and cofilin-1 mitochondrial translocation in GC. Cofilin-1, a 19-kDa ubiquitous actin-modulating protein, exists in two states, activated (cofilin-1) and inactivated (p-cofilin-1) (Vartiainen et al., 2002). The de-phosphorylation of p-cofilin-1 can induce degradation of F-actin to G-actin and promote the translocation of cofilin-1 to the mitochondria, triggering mitochondrial apoptosis (Tang et al., 2016). Many studies demonstrated that high-expression levels of cofilin-1 in many cancers correlated with tumor metastasis, chemotherapy resistance, and poor prognosis (Zhang Y. et al., 2015; Wang et al., 2019). In our previous studies, we have demonstrated that activation of PI3K/Akt can reduce the expression of the phosphoric acid lipases, PP1, PP2A, and SSH, then activate the de-phosphorylation of p-cofilin-1 and mitochondrial translocation of cofilin-1 and finally activate mitochondrial damage and induce the mitochondrial apoptosis of cisplatin-resistant gastric cancer cells (Tang et al., 2016). Previous studies demonstrated that changes in cofilin-1 or p-cofilin-1 patterns played an important role in multidrug resistance in tumor cells (Zhang et al., 2012; Li et al., 2013). All the findings demonstrated that cisplatin-resistant gastric cancer cells communicate with sensitive cells through the delivery key exosomal protein RPS3 and the activation of PI3K-Akt-cofilin-1 signaling pathway.

## Conclusion

In summary, our present study demonstrated that RPS3 protein expression levels were significantly elevated in SGC7901R cells, which was selectively sorted into SGC7901R cell-derived exosomes. Exosomal delivery of RPS3 protein may induce chemoresistance phenotypes from cisplatin-resistant gastric cancer cells to sensitive cancer cells by regulating the PI3K-Akt-cofilin-1 signaling pathway. Therefore, exosomal RPS3 protein in cisplatin-resistant gastric cancer cells may thus be a promising strategy to overcome cisplatin resistance in gastric cancer.

## Data Availability Statement

The raw data supporting the conclusions of this article will be made available by the authors, without undue reservation.

## Ethics Statement

The animal study was reviewed and approved by Animal Experimentation Ethics Committee of Shanghai University of Traditional Chinese Medicine.

## Author Contributions

Q-FT, HZ, and SC were responsible for designing the experiments and research supervision. M-YS, BX, and Q-XW were responsible for conducting experiments, acquisition of data, and analysis. SC and W-LC carried out the statistical analysis. M-YS and Q-XW wrote the manuscript. All authors reviewed and approved the final version.

## Conflict of Interest

The authors declare that the research was conducted in the absence of any commercial or financial relationships that could be construed as a potential conflict of interest.
