# Supplementary material for: Cisplatin-Resistant Gastric Cancer Cells Promote the Chemoresistance of Cisplatin-Sensitive Cells via the Exosomal RPS3-Mediated PI3K-Akt-Cofilin-1 Signaling Axis
Source: Front Cell Dev Biol. 2021 Feb 11;9:618899. doi: 10.3389/fcell.2021.618899 (PMC7905060; doi:10.3389/fcell.2021.618899)
Supplement: Supplementary file 1 [file Data_Sheet_1.docx]

**Supplementary Figures and figures legends**

**Figure S1**


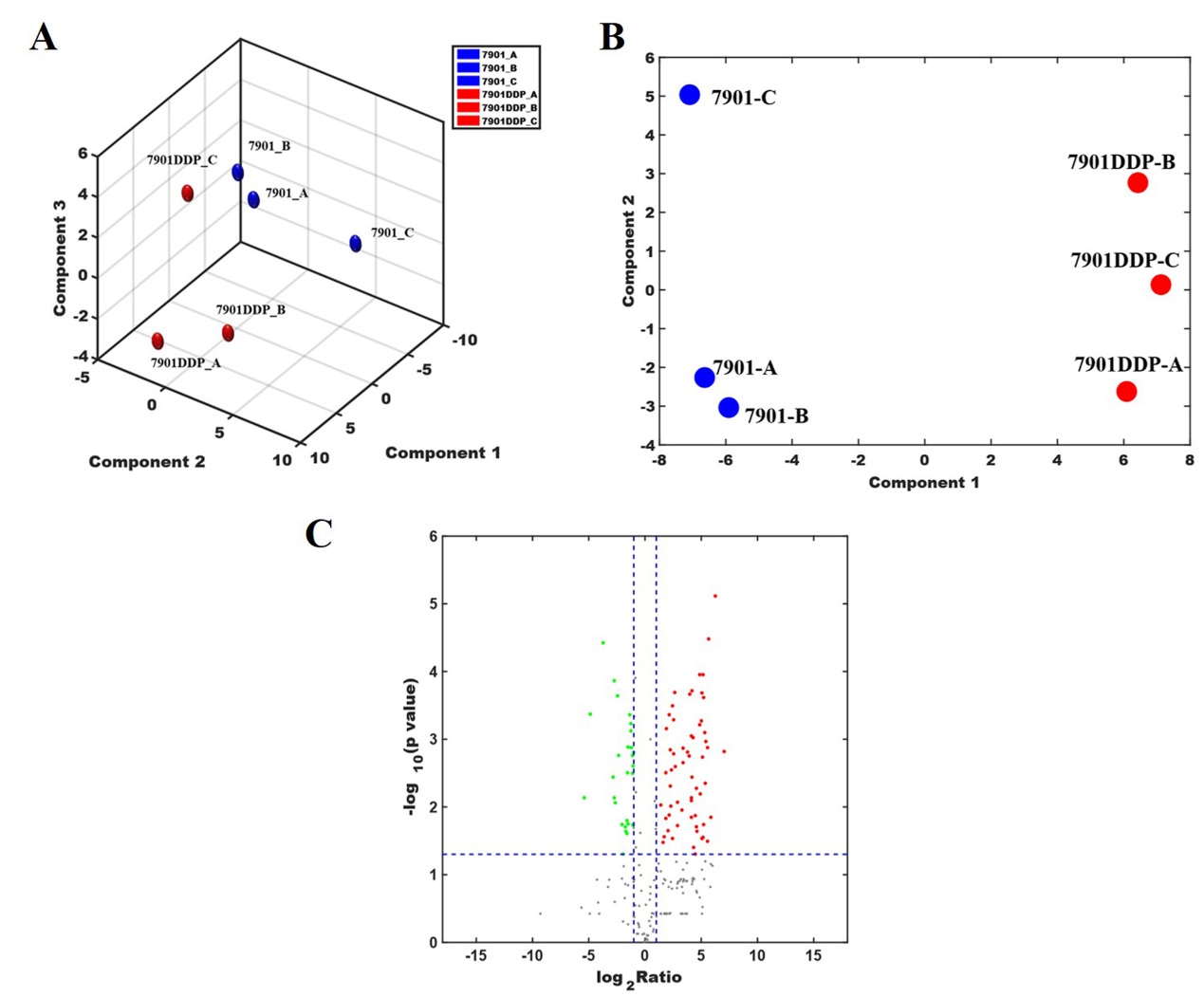
Figure S1. Principal Component Analysis (PCA) for the differentially expressed proteins between SGC7901R cells exosomes and SGC7901S cells exosomes that detected by LC-MS/MS. (A)PCAchart. (B) PCAchart (two dimensional graph). (C) Differential protein screening volcano graph. The X-axis represents the ratio of protein in the experimental group and the control group, and the Y-axis represents the p value of the repeated test results, and each point in the figure represents a protein. Red areas are up-regulated proteins and green areas are down-regulated proteins.

**Figure S2**

**
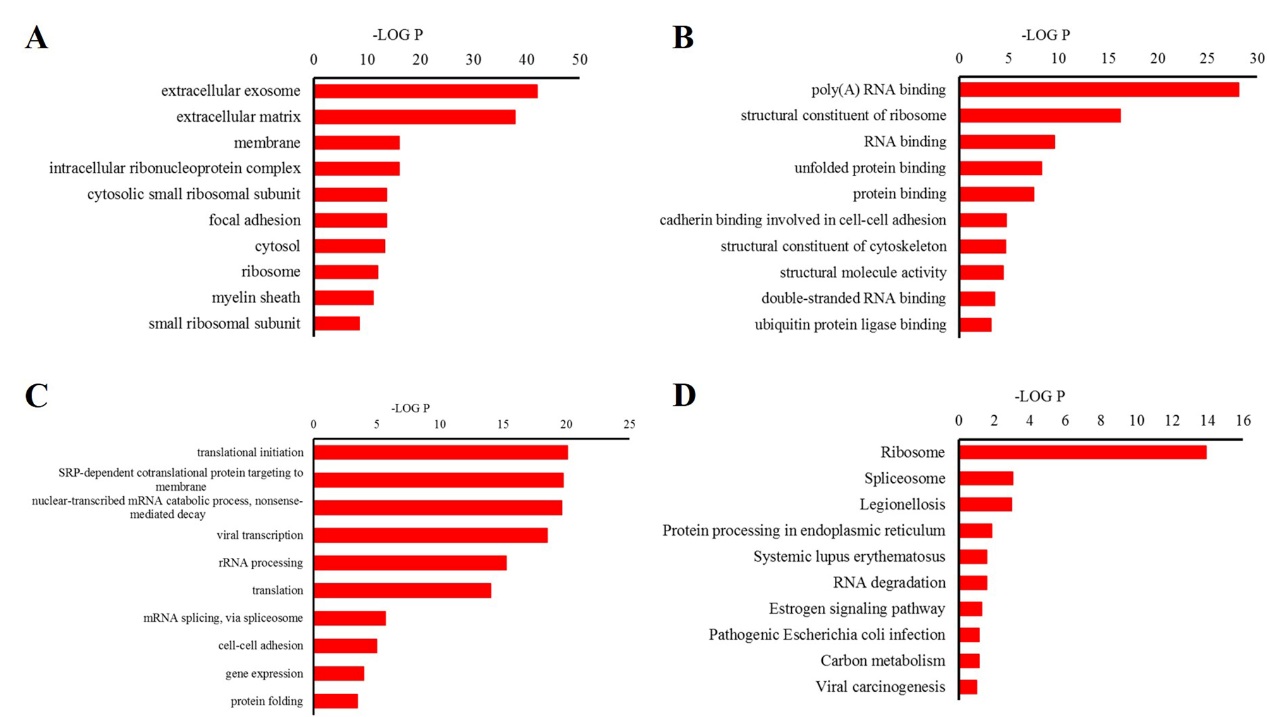
**

Figure S2. GO (Gene Ontology) and KEGG analysis for the differentially expressed proteins between SGC7901R cells exosomes and SGC7901S cells exosomes that detected by LC-MS/MS. (A) 10 subcellular localization entries with the highest number of differential proteins. (B) 10 molecular function entries with the highest number of differential proteins. (C) 10 biological process items with the highest number of differential proteins. (D) The main signaling pathway to the enrichment of differential proteins.

**Figure S3**


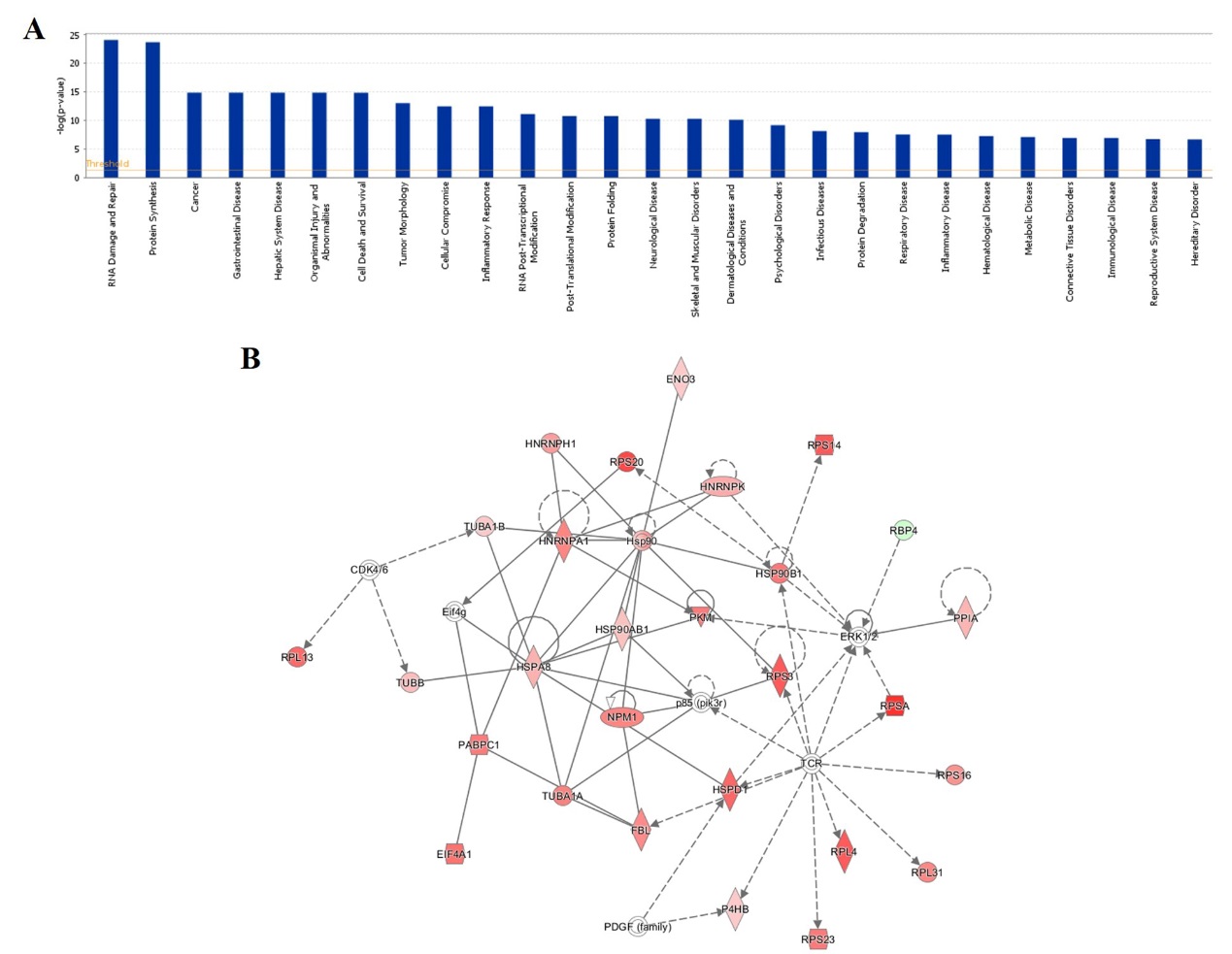


Figure S3. Functional enrichment analysis and network analysis for the differentially expressed proteins between SGC7901R cells exosomes and SGC7901S cells exosomes that detected by LC-MS/MS. (A) Functional enrichment analysis for the differentially expressed proteins. (B) Protein Synthesis, RNA Damage and Repair, Cancer related function network diagram.Red means up-regulation, and green means down-regulation.

**Figure S4**


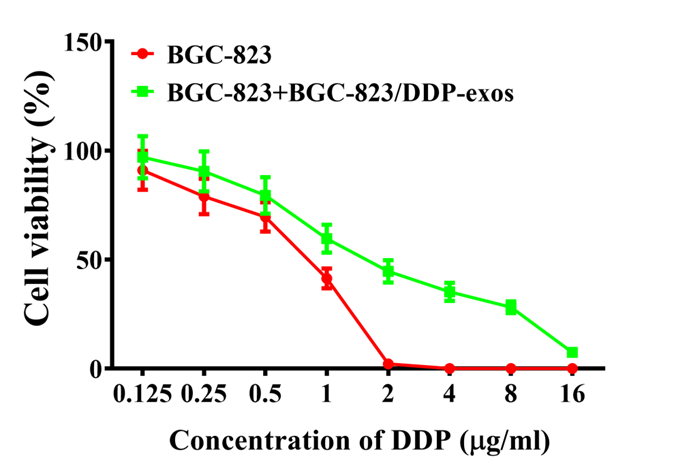


Figure S4. CCK-8 assay was performed to observe the effect of cisplatin (DDP) on the proliferation of indicated cells (BGC-823S cells or BGC-823S cells pre-treated with exosomes derived from BGC823R cells). The concentrations of cisplatin (DDP) used for the drug dose response curve analysis of indicated cells was 0, 125, 250, 500, 1000, 2000, 4000, 8000, and 16000 μg/L. Each experiment was performed in triplicate. All the data are shown as mean ± SD.

**Figure S5**


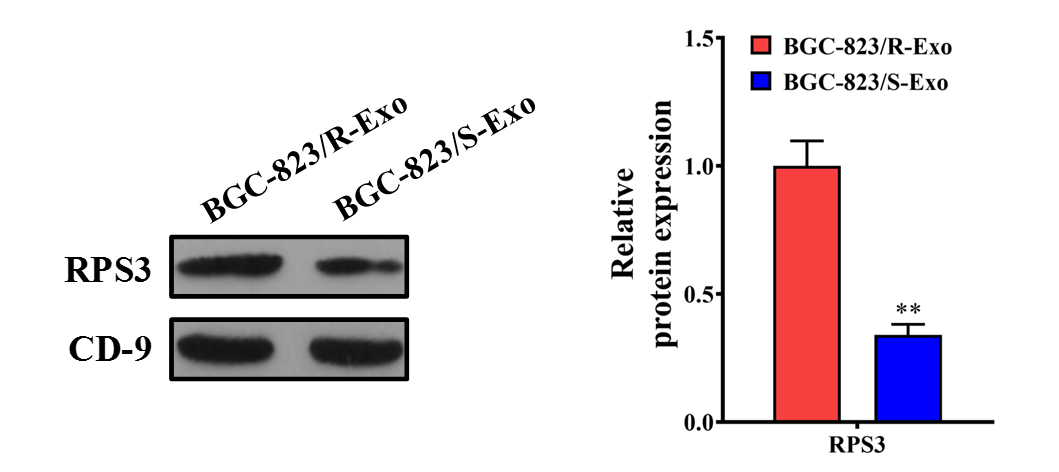


Figure S5. RPS3 protein levels were evaluated inthe exosomes of BGC823R and BGC823S cells.

**Figure S6**

**
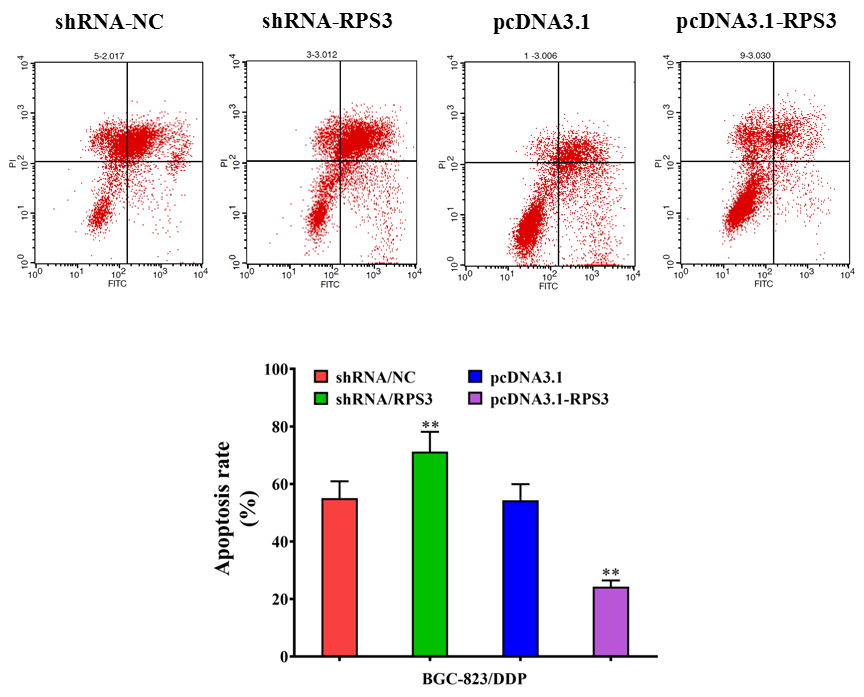
**

Figure S6. Flow cytometry method and quantitative assay were performed to observe the change of cell apoptosis in BGC823S cells treated with exosomes derived from RPS3-overexpressing or -silencing BGC823R cells, or their respective controls.

**Figure S7**


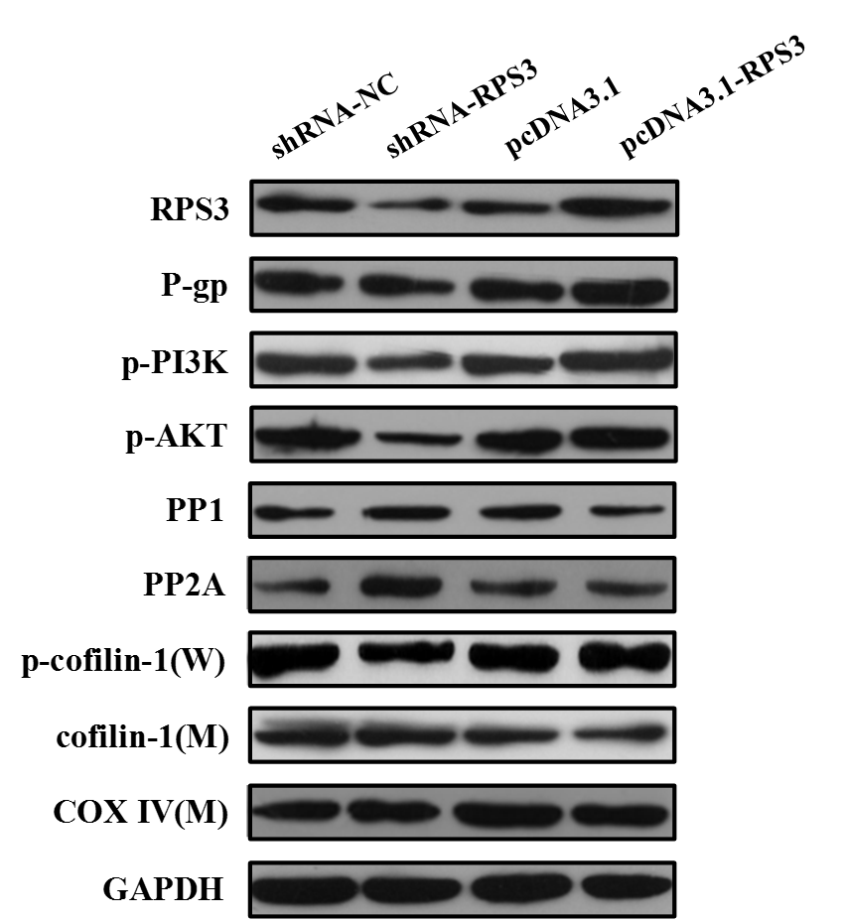


Figure S7. Western blotting and quantitative assays of p-PI3K, p-Akt, PP1, PP2A, p-cofilin-1, and cofilin-1 proteins in the BGC823S cells treated with exosomes derived from RPS3-overexpressing or -silencing BGC823R cells, or their respective controls.

**Figure S8**


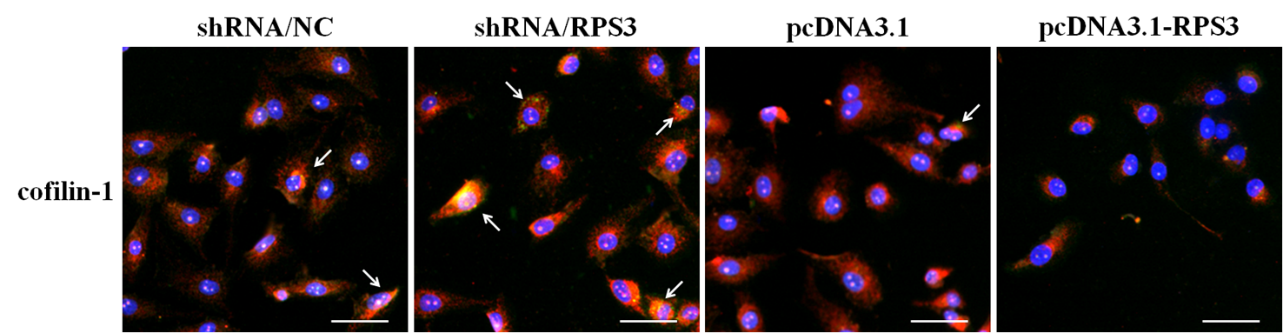


Figure S8.Immunofluorescence assay was performed to observe RPS3 overexpression inhibited translocation of cofilin-1 (green) from the cytoplasm to the mitochondria (stained with Mitotracker Red).

**Figure S9**


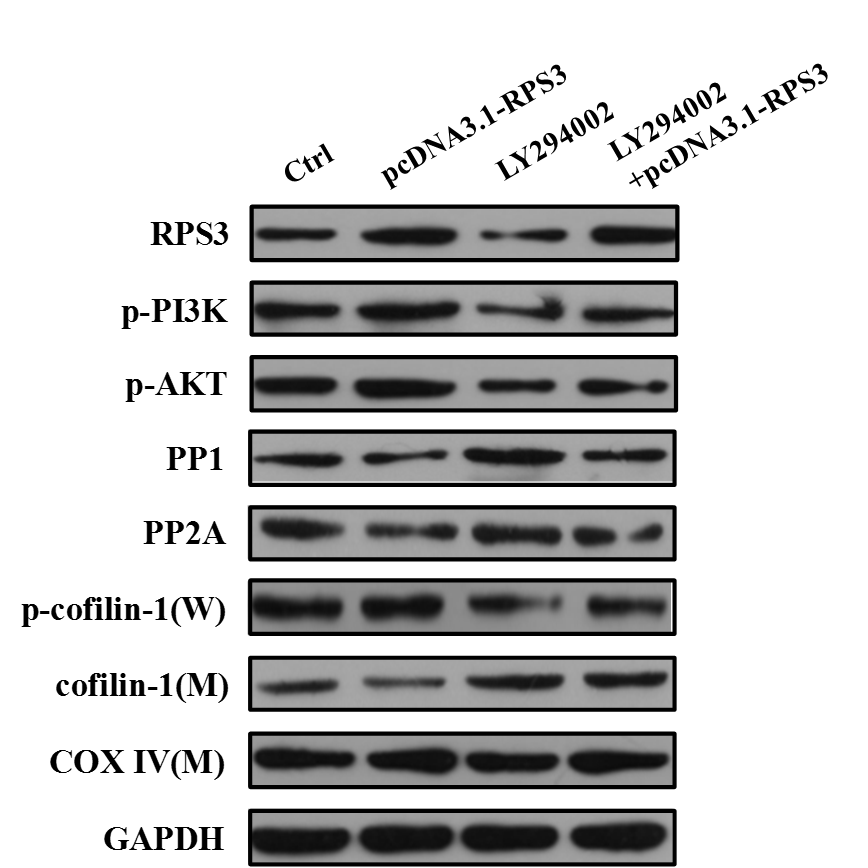


Figure S9. Cells overexpression of RPS3 were treated with or without PI3K/Akt inhibitor LY294002 for 24h, western blotting was used to detection expression levels of proteins involved in PI3K-Akt-cofilin-1 pathways.
